# Trends in aortic aneurysm- and dissection-related mortality in the state of São Paulo, Brazil, 1985–2009: multiple-cause-of-death analysis

**DOI:** 10.1186/1471-2458-12-859

**Published:** 2012-10-10

**Authors:** Augusto Hasiak Santo, Pedro Puech-Leão, Mariana Krutman

**Affiliations:** 1 Departamento de Epidemiologia, Faculdade de Saúde Pública da Universidade de São Paulo, São Paulo, Brazil; 2 Departamento de Cirurgia, Faculdade de Medicina da Universidade de São Paulo, São Paulo, Brazil; 3 Departamento de Cirurgia, Faculdade de Medicina da Universidade de São Paulo, São Paulo, Brazil

**Keywords:** Aortic aneurysm and dissection, Mortality, Multiple-cause-of-death, Historical trends, Seasonality

## Abstract

**Background:**

Aortic aneurysm and dissection are important causes of death in older people. Ruptured aneurysms show catastrophic fatality rates reaching near 80%. Few population-based mortality studies have been published in the world and none in Brazil. The objective of the present study was to use multiple-cause-of-death methodology in the analysis of mortality trends related to aortic aneurysm and dissection in the state of Sao Paulo, between 1985 and 2009.

**Methods:**

We analyzed mortality data from the Sao Paulo State Data Analysis System, selecting all death certificates on which aortic aneurysm and dissection were listed as a cause-of-death. The variables sex, age, season of the year, and underlying, associated or total mentions of causes of death were studied using standardized mortality rates, proportions and historical trends. Statistical analyses were performed by chi-square goodness-of-fit and H Kruskal-Wallis tests, and variance analysis. The joinpoint regression model was used to evaluate changes in age-standardized rates trends. A p value less than 0.05 was regarded as significant.

**Results:**

Over a 25-year period, there were 42,615 deaths related to aortic aneurysm and dissection, of which 36,088 (84.7%) were identified as underlying cause and 6,527 (15.3%) as an associated cause-of-death. Dissection and ruptured aneurysms were considered as an underlying cause of death in 93% of the deaths. For the entire period, a significant increased trend of age-standardized death rates was observed in men and women, while certain non-significant decreases occurred from 1996/2004 until 2009. Abdominal aortic aneurysms and aortic dissections prevailed among men and aortic dissections and aortic aneurysms of unspecified site among women. In 1985 and 2009 death rates ratios of men to women were respectively 2.86 and 2.19, corresponding to a difference decrease between rates of 23.4%. For aortic dissection, ruptured and non-ruptured aneurysms, the overall mean ages at death were, respectively, 63.2, 68.4 and 71.6 years; while, as the underlying cause, the main associated causes of death were as follows: hemorrhages (in 43.8%/40.5%/13.9%); hypertensive diseases (in 49.2%/22.43%/24.5%) and atherosclerosis (in 14.8%/25.5%/15.3%); and, as associated causes, their principal overall underlying causes of death were diseases of the circulatory (55.7%), and respiratory (13.8%) systems and neoplasms (7.8%). A significant seasonal variation, with highest frequency in winter, occurred in deaths identified as underlying cause for aortic dissection, ruptured and non-ruptured aneurysms.

**Conclusions:**

This study introduces the methodology of multiple-causes-of-death to enhance epidemiologic knowledge of aortic aneurysm and dissection in São Paulo, Brazil. The results presented confer light to the importance of mortality statistics and the need for epidemiologic studies to understand unique trends in our own population.

## Background

An aortic aneurysm refers to any abnormal bulging or swelling in a segment of the aorta, usually representing a primary weakness in the wall of the aorta at that location. A greater concern is the risk of rupture, which causes massive internal hemorrhage and, without prompt treatment, death occurs rapidly.

An aortic dissection is a serious progressive condition that occurs when a tear in the inner layer (intima) of the aorta causes blood to flow between the layers of the wall of the aorta and force them apart. If the blood-filled channel ruptures through the outside aortic wall, aortic dissection is usually fatal.

Prevalence, fatality and mortality rates of aortic aneurysm and dissection varies geographically. In Brazil there are only prevalence studies related to abdominal aortic aneurysms. By means of population screening, in Porto Alegre (1987 through 1993) the prevalence was 1.68% among men aged 54 years and over; in São Paulo (1999) 4.56% and 0.59% respectively among men and women aged 50 years and over, and in Vitória (2002 through 2003) 2.5% in the population over 60 years of age [1-3]. Also in São Paulo (1992 through 1995), the prevalence of infrarenal abdominal aneurysm was 4.5% in a population submitted to necropsy [4].

Aortic aneurysms and dissection were ranked in the Unites States of America in 2007 as the 19^th^ leading underlying cause-of-death for all ages [5] and as the 15^th^ leading underlying cause for those aged 65 years and over [6], according to the "List of 113 Selected Causes of Death" prepared by the National Center for Health Statistics (NCHS) [7]. To the best of our knowledge, from 1987 until now, few studies on population-based mortality trends related to aortic aneurysms and dissection have been published [8-15]. Mortality trends (1951 through 1981) in the United States showed a constant increase for all aortic aneurysms in age-adjusted rates from 1951 to 1968 and slight decline after 1968 [8]. Another report from the United States (1979 through 1992) disclosed slight variations for age-adjusted rates for dissecting and abdominal aneurysms, higher in blacks than whites and in males than females [11]. A study in England and Wales (1950 through 1984) found a 20-fold rise in age-standardized mortality rates in men and 11-fold in women, mainly due to more deaths from abdominal aneurysms [9]. Another report from England and Wales (1979 though 1999), disclosed that the number of deaths related to aneurysm as an underlying cause was increased by 43.5%, being the greatest increase observed for abdominal aortic aneurysm, where the number of deaths doubled [13]. In Japan (1955 through 1990) a 24.8-fold of increasing trends in overall age-standardized rates for males and 18.6-fold for females was observed; abdominal aortic aneurysm was the most common type, followed by dissecting and thoracic aneurysms [10]. In Australia (1968 though 1997), overall age-standardized rates remained constant for the period between 1968 and 1992 followed by a small reduction persisting until 1997. Increases in rates for abdominal and thoracic aortic aneurysms and declines for dissecting aortic aneurysms were disclosed [12]. In Scotland (1981 through 2000), age-adjusted mortality rates for thoracic aortic aneurysm increased fourfold, little change occurred with aortic aneurysms of unspecified site rates, dissecting aortic aneurysms rates remained fairly constant, and abdominal aortic aneurysms (responsible for 42.3% of all deaths), increased 2.6-fold the age-adjusted rates [14]. In England (1996 through 2004), a 3.4% decrease in the annual percentage of change for age-standardized mortality rates based on all mentions in death certificates of aortic aneurysm and dissection was observed [15].

The use of multiple-cause-of death methodology is considered necessary to provide additional useful information to the conventional underlying cause-of-death presented in primary mortality statistics [8,11,13,16]. All causes of death listed in the International Form of Medical Certificate of Cause of Death [17] are taken into account. Multiple-cause-of-death statistics are available in Brazil since 1999 and in the state of São Paulo since 1983 [18]. In 2009 there were 7,143 deaths related to aortic aneurysm and dissection in Brazil, of which 6,057 (84.80%) were listed as underlying cause and 1,086 (15.20%) as associated (non-underlying) causes of death [19].

The objective of the present study was to study trends in mortality related to aortic aneurysm and dissection in the state of São Paulo between 1985 and 2009 using multiple causes of death analysis.

## Methods

Brazil is the world's fifth largest country, both geographically and demographically, with an estimated population of 192 million people. Brazilian is the world's sixth largest economy and São Paulo, located in the southeastern region, is the most developed state among the federative units responsible for 34% of the Gross Internal Product. The Brazilian 2010 Census revealed for the state a total population of 41,262,199 with 3,324,427 (7.84%) inhabitants over 65 years of age. In 1985 and 2009, respectively, there were 198,282 and 256,627 overall deaths, and 71,738 (39.36%) and 144,635 (56.36%) occurred in the population over 65 year of age.

Annual mortality data was obtained from the public multiple-cause-of-death databases of the *Sistema Estadual de Análise de Dados* (SEADE, State Data Analysis System) Foundation, the organ responsible for drawing up vital statistics, operating under the auspices of the São Paulo State Secretary of Economics and Planning. All deaths were selected in which aortic aneurysm and dissection were listed on any line or in either part of the International Form of Medical Certificate of Cause-of-death (the medical certification section of the death certificate), irrespective of whether categorized as the underlying cause-of-death or as an associated (non-underlying) cause. Complications of the underlying cause (Part I of the medical certification section) and contributing causes (Part II of the medical certification section) were collectively designated as associated (non-underlying) causes of death. Deaths in the state of São Paulo of non-residents were excluded from the selection.

We employed the 1985–2009 mid-year estimates of the population for the state of São Paulo, discriminated by year, sex, age group and administrative health areas of the state. These estimates were recently updated by the SEADE Foundation based on recent demographic changes noted in the 2010 Brazilian Census.

We selected all deaths in which one or more rubrics of the aortic aneurysm and dissection category of the ninth or tenth revisions of the International Classification of Diseases (ICD-9 or ICD-10) were listed on death certificates [20,21]. Aortic syphilitic aneurysms, classified as underlying or associated cause-of-death, were excluded from the selection. Table 1 shows the rubrics equivalence used to evaluate the trends of the causes of death during the periods that ICD-9 and ICD-10 revisions were in force [22,23].

**Table 1:** Category and subcategories rubrics and codes related to aortic aneurysm and dissection included in the ninth and tenth revisions of the International Classification of Diseases

**Category and subcategories**	**ICD**-**9**	**ICD**-**10**
**Aortic aneurysm and dissection**	**441**	**I71**
Dissection of aorta [any part]	441.0	I71.0
Thoracic aortic aneurysm, ruptured	441.1	I71.1
Thoracic aortic aneurysm, without mention of rupture	441.2	I71.2
Abdominal aortic aneurysm, ruptured	441.3	I71.3
Abdominal aortic aneurysm, without mention of rupture	441.4	I71.4
Thoracoabdominal aortic aneurysm, ruptured		I71.5
Thoracoabdominal aortic aneurysm, without mention of rupture		I71.6
Aortic aneurysm of unspecified site, ruptured	441.5	I71.8
Aortic aneurysm of unspecified site, without mention of rupture	441.6	I71.9

In order to reconstruct the morbid process leading to death, all causes of death listed in the medical certification section of the death certificate were considered, including those classified as ill-defined or characterized by the World Health Organization (WHO) as modes of death [17].

Records included in the mortality databases contain fields that mirror those items appearing on official Brazilian death certificate. In the present study, it was necessary to standardize the structure of the records studied, since various fields on the death certificate underwent changes in designation, size and codes between 1985 and 2009. In addition, we created auxiliary fields for the study of multiple causes, including a field designed to contain a single "string" of characters composed of the codes entered on lines (a), (b), (c) and (d) of Parts I and of Part II of the medical certification section of the death certificate.

The causes of death had been automatically processed with the softwares *Automated Classification of Medical Entities* (ACME) between 1985 and 1995 [24-29], and *Declarações de Óbito de São Paulo* (DOSP, São Paulo Death Certificates) (between 1996 and 2009) [30], an adaptation that allowed the data to be processed in batches using the *Underlying Cause Selection* program [26]. The data regarding the associated causes of death in 1996 are incomplete due to the fact that the SEADE Foundation implemented the use of the DOSP in April of that year. The automatic processing involves the use of algorithms and decision tables that incorporate the WHO mortality standards and the etiological relationships among the causes of death. The ACME decision tables [31,32] are considered *de facto* international standards for the automatic processing of data related to the causes of death [33]. The structural difference between the numeric codes employed in the ICD-9 (used up through 1995) and the alphanumeric codes employed in the ICD-10 (in use since 1996) affected the processing of the data in that a different software was required for each coding system. As a consequence, we studied the trends in mortality related to aortic aneurysm and dissection in two separate periods: 1985–1995 and 1996–2009. The expressions " death from" and " death due to" refer to the underlying cause-of-death, whereas " deaths with a mention of" and " mortality related to" refer to the listing of a given condition either as the underlying cause or as an associated cause. The causes of death evaluated in the present study were those actually mentioned in the medical certification section, which are known internationally as “*entity axis codes”*, defined and presented under the structure and headings of the ICD [24,34].

Using mortality rates, proportions and historical trends, we studied the distributions of the following variables: sex, age at death (in five year age-groups), year of death, underlying cause-of-death, associated (non-underlying) cause(s) of death, total mentions of each cause-of-death, mean number of causes listed per death certificate, seasonal variation of deaths and geographical distribution of deaths in the state of São Paulo. For the purpose of seasonal analysis, deaths were grouped as follows: summer, December 21^st^ though March 20^th^; autumn, March 21^st^ through June 20^th^; winter, June 21^st^ through September 20^th^ and spring, September 21^st^ through December 20^th^. Medical and demographic variables were processed with the following softwares: dBASE III Plus, version 1.1 (Ashton-Tate Corporation, Torrance, CA), Epi Info, version 6.04d (Centers for Disease Control and Prevention, Atlanta, GA), Excel 2007 (Microsoft Corporation, Redmond, WA) and BioEstat 5.0 *Aplicações estatísticas nas areas bio-médicas* (Statistical applications in biomedical areas) [35]. We used the *Tabulador de Causas Múltiplas* for Windows (Multiple Causes Tabulator) program (DATASUS, Ministério da Saúde, Faculdade de Saúde Pública, Universidade de São Paulo, Brazil), processing codes for ICD-9 and ICD-10 (TCMWIN, version 1.6), in our presentation of the associated causes and of the mean number of causes per death certificate [36].

For the presentation of the associated causes listed on the death certificates on which aortic aneurysm and dissection were identified as the underlying cause, we prepared special lists showing the causes involved in the respective natural histories [37-41], as well as those mentioned with the greatest frequency. The duplication or multiplication of causes of death was avoided when these were presented in abbreviated lists. The number of causes depends on the breadth of the class (subcategory, category, grouping or chapter of the ICD-9 or ICD-10); therefore, if two or more causes mentioned in the medical certification section were included in the same class, only one cause was computed [24,36].

Mortality rates (per 100,000 population) for aortic aneurysm and dissection were calculated—by year, by subperiod and for the study period (1985–2009) as a whole—based on the number of deaths in which each disease had been identified as the underlying cause or as an associated cause, as well as on the total number of mentions of each disease. In order to calculate the mean mortality rate for each subperiod studied, the number of deaths occurring in the period was divided by the sum of the respective annual population counts, analogous to the procedure used in order to calculate the mean mortality rate for the 25-year study period as a whole.

The *Programa para Análisis Epidemiológico de Datos* (Epidat, Epidemiological Analysis of Data Program), version 4.0 (Dirección Xeral de Innovación e Xestión da Saúde Pública, Xunta de Galicia: http://dxsp.sergas.es, and Pan American Health Organization) was used for standardize, by the direct method, the age-adjusted crude and mean mortality rates, by subperiod and for the study period as a whole, to the new WHO Standard Population [42].

### Statistical analysis

We used analysis of variance to compare the mean numbers of causes mentioned on the death certificate; the Kruskal-Wallis H test to compare the mean age at death between groups; and chi-square goodness-of-fit test to analyze the uniformity within the seasonal distribution of aortic aneurysms and dissection deaths. The joinpoint regression model was used to evaluate the changes in age-standardized rates trends [43]. Assuming a Poisson distribution, joinpoint analysis chooses the best fitting point (or points), at which the rate increase or decrease significantly. For analysis uniformity and synthesis, we allowed one joinpoint. Values of p < 0.05 were considered significant.

## Results

Over the time period from 1985 to 2009, among residents of the state of São Paulo, 42,615 deaths related to dissection and aortic aneurysms occurred, of which 36,088 (84.7%) were identified to be the underlying cause and 6,527 (15.3%) an associated (non-underlying) cause-of-death, corresponding, respectively, to the averaged age-standardized death rates of 6.58, 5.53 and 1.05 deaths per 100,000 population. Considered as underlying causes of death, dissection (37.0%), ruptured abdominal aortic aneurysm (17.3%) and ruptured aortic aneurysm of unspecified site (17.5%) were most frequent, whereas as an associated (non-underlying) cause abdominal aortic aneurysm without mention of rupture (35.4%) and aortic aneurysm of unspecified site without mention of rupture (22.2%) prevailed (Table 2).

**Table 2:** Mortality related to aortic aneurysm and dissection, according to the qualification of the cause-of-death and sex, age-standardized death rates, absolute numbers and percentage of aneurysmal types and of mention of rupture, age at death and number of causes of death per death certificate, state of São Paulo, Brazil, 1985 to 2009

**Variables**	**Underlying**			**Associated** (**Non Underlying**)			**Mentions**		
	**Men**	**Women**	**Subtotal**	**Men**	**Women**	**Subtotal**	**Men**	**Women**	**Total**
AGE STANDARDIZED DEATH RATES PER 100.000 POPULATION									
In 1985	5.34	2.01	3.56	0.94	0.18	0.52	6.28	2.20	4.08
In 2009	8.74	4.10	6.09	1.60	0.62	1.02	10.35	4.72	7.12
Average age-standardized rates from 1985 through 2009	8.23	3.42	5.53	1.71	0.56	1.05	9.94	3.98	6.58
Rates Ratio 2009/1985	1.64	2.04	1.71	1.70	3.37	1.98	1.65	2.15	1.75
AORTIC ANEURYSM AND DISSECTION TYPES (ICD-9) (ICD-10). ABSOLUTE NUMBERS (%)									
Dissection of aorta [any part] (441.0) (171.0)	8,056 (57.2)	5,304 (37.7)	13,360 (94.8)	456(3.2)	272(1.9)	728 (5.2)	8,512(60.4)	5,576 (39.60	14,088 (100.0)
	(34.1)	(42.7)	(37.4)	(10.1)	(13.5)	(11.2)	(30.2)	(38.6)	(33.1)
Thoracic aortic aneurysm, ruptured (441.1) (171.1)	1,732 (54.6)	1,262 (39.8)	2,994 (94.3)	108 (3.4)	72 (2.3)	180 (5.7)	1,840 (58.0)	1,334 (42.0)	3,174 (100.0)
	(7.3)	(10.2)	(8.3)	(2.4)	(3.6)	(2.8)	(6.5)	(9.2)	(7.5)
Thoracic aortic aneurysm, without mention of rupture (441.2) (171.2)	751 (37.9)	489 (24.7)	1,240 (62.6)	427 (21.6)	314 (15.9)	741 (37.4)	1,178 (59.5)	803 (40.5)	1,981 (100.0)
	(3.2)	(3.9)	(3.4)	(9.5)	(15.6)	(11.4)	(4.2)	(5.6)	(4.7)
Abdominal aortic aneurysm, ruptured (441.3) (171.3)	4,860 (72.2)	1,399 (20.8)	6,259 (92.9)	365 (5.4)	112 (1.7)	477 (7.1)	5,225 (77.6)	1,511 (22.4)	6,736 (100.0)
	(20.5)	(11.3)	(17.3)	(8.1)	(5.6)	(7.3)	(18.6)	(10.5)	(15.8)
Abdominal aortic aneurysm, without mention of rupture (441.4) (171.4)	2,363 (43.7)	735 (13.6)	3,098 (57.3)	1,753 (32.4)	560 (10.4)	2,313 (42.8)	4,116 (76.1)	1,295 (23.9)	5,411 (100.0)
	(10.0)	(5.9)	(8.6)	(38.9)	(27.8)	(35.4)	(14.6)	(9.0)	(12.7)
Thoracoabdominal aortic aneurysm, ruptured (171.5)	150 (56.6)	97 (36.6)	247 (93.2)	12 (4.5)	6 (2.3)	18 (6.8)	162 (61.1)	103 (38.9)	265 (100.0)
	(0.6)	(0.8)	(0.7)	(0.3)	(0.3)	(0.3)	(0.6)	(0.7)	(0.6)
Thoracoabdominal aortic aneurysm, without mention of ruptured (171.6)	166 (41.0)	108 (26.7)	274 (67.7)	77 (19.0)	54 (13.3)	131 (32.4)	243 (60.0)	162 (40.0)	405 (100.0)
	(0.7)	(0.9)	(0.8)	(1.7)	(2.7)	(2.0)	(0.9)	(1.1)	(1.0)
Aortic aneurysm of unspecified site, ruptured (441.5) (171.8)	4,015 (58.9)	2,316 (34.0)	6,331 (92.8)	326 (4.8)	164 (2.4)	490 (7.2)	4,341 (63.6)	2,480 (36.4)	6,821 (100.0)
	(17.0)	(18.6)	(17.5)	(7.2)	(8.1)	(7.5)	(15.4)	(17.2)	(16.0)
Aortic aneurysm of unspecified site, without mention of rupture (441.6) (171.9)	1,565 (41.9)	720 (19.3)	2,285 (61.2)	986 (26.4)	463 (12.4)	1,449 (38.8)	2,551 (68.3)	1,183 (31.7)	3,734 (100.0)
	(6.6)	(5.8)	(6.3)	(21.9)	(23.0)	(22.2)	(9.1)	(8.2)	(8.8)
Total	23,658 (55.5)	12,430 (27.2)	36,088 (84.7)	4,510 (10.6)	2,017 (4.7)	6,527 (15.3)	28,168 (66.1)	14,447 (33.9)	42,615 (100.0)
	(100.0)	(100.0)	(100.0)	(100.0)	(100.0)	(100.0)	(100.0)	(100.0)	(100.0)
AORTIC ANEURYSM AND DISSECTION WITH MENTION OF RUPTURE (ICD-9) (ICD-10). ABSOLUTE NUMBERS (%)									
Dissecting (441.0) (171.0)	8,056 (57.2)	5,304 (37.7)	13,360 (94.8)	456 (3.2)	272 (1.9)	728 (5.2)	8,512 (60.4)	5,576 (39.6)	14,088 (100.0)
	(34.1)	(42.7)	(37.0)	(10.1)	(13.5)	(11.2)	(30.2)	(38.6)	(33.1)
Ruptured (441.1) (171.1) (441.3) (171.3) (171.5) (441.5) (171.8)	10,757 (63.3)	5,074 (29.9)	15,831 (93.2)	811 (4.8)	354 (2.1)	1,165 (6.9)	11,568 (68.1)	5,428 (31.9)	16,996 (100.0)
	(45.5)	(40.8)	(43.9)	(18.0)	(17.6)	(17.9)	(41.1)	(37.6)	(39.9)
Without rupture (441.2) (171.2) (441.4) (171.4) (171.6) (441.6) (171.9)	4,845 (42.0)	2,052 (17.8)	6,897 (59.8)	3,243 (28.1)	1,391 (12.1)	4,634 (40.2)	8,088 (70.1)	3,443 (29.9)	11,531 (100.0)
	(20.5)	(16.5)	(19.1)	(71.9)	(69.0)	(71.0)	(28.7)	(23.8)	(27.1)
Total	23,658 (55.5)	12,430 (27.2)	36,088 (84.7)	4,510 (10.6)	2,017 (4.7)	6,527 (15.3)	28,168 (66.1)	4,447 (33.9)	42,615 (100.0)
	(100.0)	(100.0)	(100.0)	(100.0)	(100.0)	(100.0)	(100.0)	(100.0)	(100.0)
AORTIC ANEURYSM AND DISSECTION WITH MENTION OF RUPTURE (ICD-9) (ICD-10), MEAN AGES AT DEATH AND STANDARD DEVIATION (IN YEARS)									
Dissecting (441.0) (I71.0)	61.4 (±13.6)	65.7 (±14.0)	63.1 (±13.9)	63.4 (±14.5)	66.3 (±14.8)	64.5 (±14.6)	61.5 (±13.7)	65.8 (±14.0)	63.2 (±14.0)
Ruptured (441.1) (I71.1) (441.3) (I71.3) (I71.5) (441.5) (I71.8)	67.4 (±13.2)	70.2 (±14.1)	68.3 (±13.5)	69.6 (±13.0)	70.9 (±16.0)	70.0 (±14.0)	67.6 (±13.2)	70.2 (±14.2)	68.4 (±13.6)
Without rupture (441.2) (I71.2) (441.4) (I71.4) (I71.6) (441.6) (I71.9)	69.4 (±11.8)	71.4 (±13.4)	70.0 (±12.3)	73.1 (±11.2)	76.4 (±12.0)	74.1 (±11.5)	70.9 (±11.7)	73.5 (±13.1)	71.6 (±12.2)
Total	65.8 (±13.4)	68.5 (±14.1)	66.7 (±13.8)	71.5 (±12.3)	74.1 (±13.7)	72.3 (±12.8)	66.7 (±13.4)	69.3 (±14.2)	67.6 (±13.8)
AORTIC ANEURYSM AND DISSECTION MORTALITY STUDY PERIODS, MEAN NUMBER OF CAUSES OF DEATH PER DEATH CERTIFICATE AND STANDARD DEVIATION									
1985-1995 (ICD-9)	2.94 (±1.11)	2.9 (±1.08)	2.93 (±1.10)	4.06 (±1.07)	4.01 (±1.09)	4.05 (±1.07)	3.1 (±1.17)	3.04 (±1.14)	3.08 (±1.17)
1996-2009 (ICD-10)	3.38 (±1.35)	3.26 (±1.30)	3.33 (±1.33)	4.49 (±1.17)	4.46 (±1.17)	4.48 (±1.17)	3.56 (±1.38)	3.43 (±1.35)	3.52(±1.37)

Source: Fundação Sistema Estadual de Análise de Dados -FSEADE.

**Figure 1:**
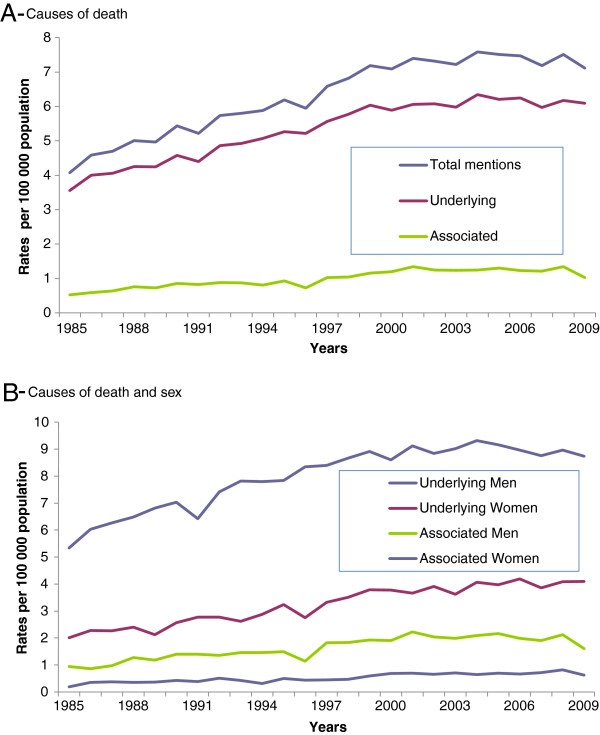
Trends in annual age-standardized death rates related to aortic aneurysm and dissection by causes of death in A and causes of death and sex in B, state of São Paulo, 1985–2009.

For the entire period of 1985 to 2009, a significant increased trend of age-standardized death rates was established in joinpoint regression analysis for aortic aneurysm and dissection as causes of death for all mentions, underlying cause, and associated (non-underlying) in men and women (Table 3 and Figure 1A and 1B), whose corresponding averaged rates for the entire period are displayed in Tables 2 and 3. This significant increased trend was evident from 1985 to 1999/2002, when a certain levelling off and even decreasing trend was observed, however non-statistically significant.

**Table 3:** Aortic aneurysm and dissection trends and Joinpoint Regression Analysis^**a**^by cause-of-death, sex and site, State of São Paulo, 1985 to 2009

**Aortic aneurysm and dissection variables**	**Global 1985-2009**			**Trend 1**			**Trend 2**		
	**Rates**^**b**^	**APC**	**CI**	**Years**	**APC**	**CI**	**Years**	**APC**	**CI**
CAUSES OF DEATH									
Causes of death (refer to Figure1A)									
All mentions	6.58	2.1^c^	1.7 to 2.5	1985-2001	3.3^c^	2.9-to 3.7	2001-2009	−0.3	−1.0 to 0.5
Underlying cause-of-death	5.53	2.0^c^	1.6 to 2.3	1985-1999	3.4^c^	3.0 to 3.8	1999-2009	0.3	−0.1 to 0.8
Associated cause-of-death	1.05	2.9^c^	1.8 to 4.0	1985-2002	4.7^c^	3.5 to 5.9	2002-2009	−1.3	−4.1 to 1.6
Causes of death and sex (refer to Figure1B)									
Underlying cause-of-death in men	8.23	1.7^c^	1.3 to 2.1	1985-1999	3.2^c^	2.7 to 3.7	1999-2009	−0.1	−0.7 to 0.5
Underlying cause-of-death in women	3.42	2.8^c^	2.4 to 3.3	1985-2000	4.1^c^	3.2 to 5.0	2000-2009	1.1	−0.1 to 2.3
Associated cause-of-death in men	1.71	2.8^c^	1.9 to 3.7	1985-2002	4.8^c^	3.5 to 6.1	2002-2009	−2.1	−5.3 to 1.1
Associated cause-of-death in women	0.56	3.7^c^	2.8 to 4.6	1985-2009	3.7^c^	2.8 to 4.6			
AORTIC ANEURYSM AND DISSECTION SITES – ALL MENTIONS									
Total mentions (refer to Figure2A)									
Dissecting	2.09	1.3^c^	0.8 to 1.8	1985-2003	2.1^c^	1.4 to 2.8	2003-2009	−2.0	−4.5 to 0.6
Abdominal	1.96	2.4^c^	1.8 to 3.0	1985-2001	3.9^c^	3.0 to 4.8	2001-2009	−0.4	−2.0 to 1.2
Unspecified site	1.63	1.0^c^	0.3 to 1.7	1985-1996	4.5^c^	2.8 to 6.2	1996-2009	−0.9^c^	−1.9 to 0.0
Thoracic	0.79	4.8^c^	4.1 to 5.5	1985-2009	4.8^c^	4.1 to 5.5			
Total mentions in men (refer to Figure2B)									
Abdominal	3.55	2.4^c^	1.8 to 3.0	1985-2001	4.0^c^	3.1 to 4.9	2001-2009	−0.6	−2.2 to 1.1
Dissecting	2.78	0.8^c^	0.3 to 1.4	1985-2003	1.7^c^	0.9 to 2.6	2003-2009	−2.6	−5.8 to 0.8
Unspecified site	2.43	0.7^c^	0.1 to 1.5	1985-1996	4.5^c^	2.5 to 6.6	1996-2009	−1.5^c^	−2.6 to 0.3
Thoracic	1.03	4.1^c^	3.2 to 5.0	1985-2009	4.1^c^	3.2 to 5.0			
Total mentions in women (refer to Figure2C)									
Dissecting	1.51	2.0^c^	1.4 to 2.6	1985-2004	2.9^c^	2.1 to 3.7	2004-2009	−2.2	−6.0 to 1.9
Unspecified site	1.01	1.9^c^	1.2 to 2.6	1985-1997	4.4^c^	2.3 to 6.6	1997-2009	0.3	−1.1 to 1.6
Abdominal	0.79	3.2^c^	2.4 to 3.9	1985-2009	3.2^c^	2.4 to 3.9			
Thoracic	0.59	5.8^c^	5.8 to 6.6	1985-2009	5.8^c^	5.0 to 6.6			
REFERENCE OF RUPTURE - ALL MENTIONS (refer to Figure 3)									
Ruptured	2.65	3.1^c^	2.1 to 4.1	1985-1997	8.4^c^	6.7 to 10.1	1997-2009	0.0	−0.9 to 1.0
Without rupture	1.84	1.5^c^	0.7 to 2.3	1985-2009	1.5^c^	0.7 to 2.2			

APC: Annual percent change; CI: confidence interval.

^a^ Rates are per 100 000 population, age-standardized to the WHO Standard Population.

^b^ Joinpoint analysis of trends allowed for one joinpoint.

^c^ The annual percent change is statistically significantly different from 0 (p < 0.05).

### Aortic aneurysm and dissection and sex

Considering both ruptured aortic aneurysms and without mention of rupture and as its total mentions in death certificates, the main specified causes of death were dissection (33.1%), abdominal aneurysms (28.5%), aneurysm of unspecified site (24.8%) and thoracic aneurysms (12.2%); among men abdominal aortic aneurysms (33.2%), dissections (30.2%) and among women dissection (38.6%) and aortic aneurysms of unspecified site (25.4%) prevailed (Table 2).

**Figure 2:**
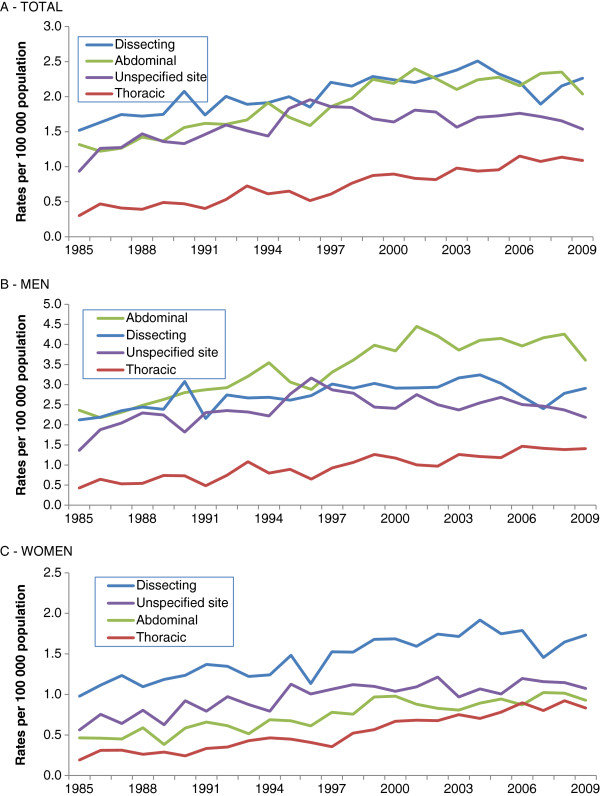
**Trends in annual age-standardized death rates related to all mentions of aortic aneurysm and dissection sites in death certificates, total in A**, **of men in B and of women in C**, **state of São Paulo**, **1985**–**2009.**

A significant increase of corresponding age-standardized death rates was also established by joinpoint regression analysis for these sites in both sexes for the entire period (Table 3 and Figure 2A, B and C). The steadily increasing trend, varying from annual percent change (APC) of 1.7% to 5.8% among aortic aneurysm sites and dissection, was also more evident from 1985 to 1996/2004, when a certain levelling off and decrease occurred, nevertheless significant only for aortic aneurysm of unspecified site for men (APC = −1.5%), which reflected in all-mentions age-standardized death rates (APC = −0.9%).

Alternatively, the ratios of all mentions age-standardized death rates from 2009 to 1985 were 1.65 for men and 2.15 for women. As corollary, the parallel death rates ratios of men to women was 2.86 for 1985 and 2.19 for 2009, corresponding to a difference decrease of 23.43% between men and women rates (Table 2).

### Aortic aneurysm and dissection and mention of rupture

**Figure 3:**
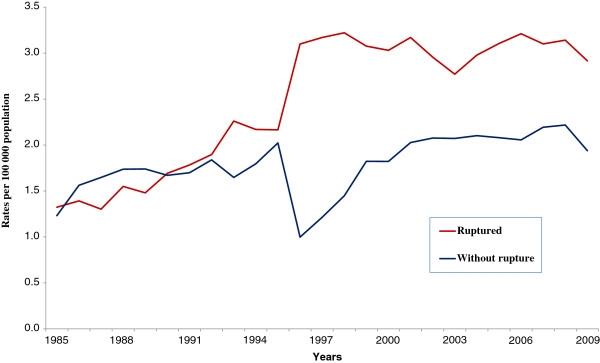
**Trends in annual age**-**standardized death rates related to all mentions of aortic aneurysm in death certificates**, **according to reference of rupture**, **state of São Paulo**, **1985**–**2009.**

Dissection and ruptured aneurysms were identified as an underlying cause in greater proportions accounting around 93% at the same time as aneurysms without mention of rupture in about 60% (Table 2). The increased trend of age-standardized mortality rates also appeared with ruptured and without mention of rupture aortic aneurysms, mainly for ruptured aneurysms with annual percent change of 8.4% from 1985 to 1997, when a non significant levelling off occurred until 2009 (Table 3). Noteworthy the marked difference with these rates was the impressive influence of the fourth line included in the International Form of Medical Certificate of Causes of Death and the ICD-10 new guidelines for the selection of the underlying cause-of-death occurred in 1996 in Brazil. It is our opinion that the fourth line gave opportunity to physicians to better inform the occurrence of rupture of aneurysms (Figure 3).

### Mean ages at death

**Figure 4:**
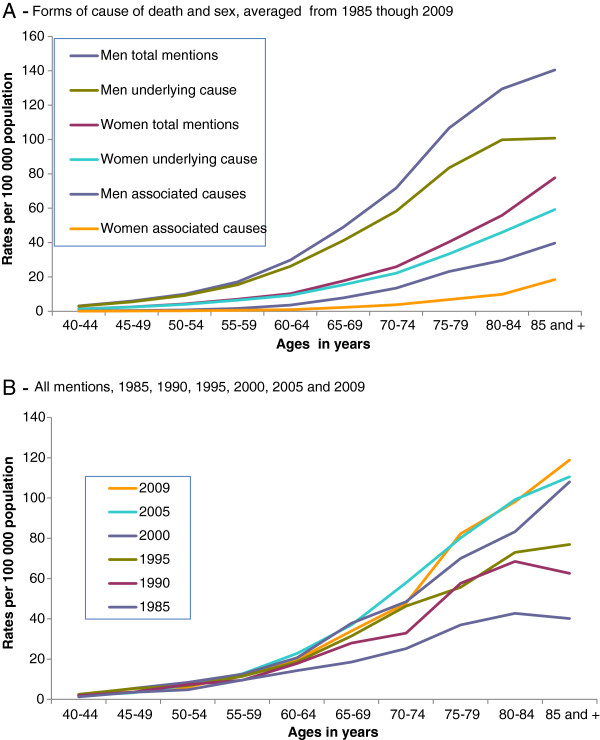
**Specific death rates related to aortic aneurysm and dissection**, **by age**, **state of São Paulo**, **1985**–**2009.**

The mean ages at death are heavily influenced by the sex of the deceased and the qualification of the cause-of-death, being constantly higher in women or when dissection and aortic aneurysm were considered as associated (non-underlying) causes. Accordingly, for the entire period and total mentions, the mean age at death of women was 69.3 (±14.2) years and of men 66.7 (±13.4) (p=0.000000), and for dissection and aortic aneurysms as an associated cause 72.3 (±12.8) years in contrast to those as an underlying cause 66.7(±13.8) years (p=000000). Regarding dissection and reference of rupture of aneurysms, the mean ages at death were steadily lower among the deaths related to the dissection of aorta (63.2±14.0 years) than among deaths related to ruptured aneurysms (68.4±13.6 years) and aneurysms without mention of rupture (71.6±12.2 years) (p=0.00000), ages considering all mentioned causes and also for the entire period (Table 2). A trend of increasing mean age at death was observed from 1985 through 2009 for the all-inclusive ICD group of dissection and aortic aneurysm, going respectively as an underlying cause (p<0.0001) from 61.7 (±14.6) years to 68.6 (±13.5) years and as an associated cause (p<0.0001) from 68.2 (16.3) years to 74.0 (±12.3) years, whose graphs for older ages are shown in Figure 4A and B.

### Associated (non-underlying) causes in the deaths related to aortic aneurysm and dissection as the underlying cause

For the 25-year study as a whole, the major associated causes on those deaths in which aortic aneurysm and dissection were identified as the underlying cause are presented in Table 4, in decreasing order of their total mentions. Hemorrhages, mentioned in death certificates roughly 40% with dissection and ruptured aneurysms, hypertensive diseases in 49% with dissection, shock (33%) and atherosclerosis (25%) with ruptured aneurysms and surgical operations and procedures (28%) and related complications (26%) with aneurysms without rupture deserve special attention. Ischemic heart diseases, renal failure, heart failure and chronic lower respiratory diseases were also important associated causes and frequent among aneurysms without rupture.

**Table 4:** Associated causes in deaths that listed aortic aneurysm and dissection selected as underlying cause-of-death, State of São Paulo, 1985-2009

**Associated** (**non**-**underlying**) **causes of death** (**ICD**-**9**) (**ICD**-**10**)	**Aortic aneurysm and dissection as underlying cause**-**of**-**death**							
	**Dissection**		**Ruptured aneurysms**		**Without ruputure**		**Total**	
	**deaths** = **13**,**360**		**deaths** = **15**,**831**		**deaths** = **6**,**897**		**deaths** = **36**,**088**	
	**n**	**%**	**n**	**%**	**n**	**%**	**n**	**%**
Haemorrhages ^a^	5,848	43.8	6,415	40.5	959	13.9	13,222	36.6
Hypertensive diseases (401–404) (I10-I13)	6,572	49.2	3,547	22.4	1,689	24.5	11,808	32.7
Schok. not elsewhere classified (785.5) (R57)	2,547	19.1	5,192	32.8	1,449	21.0	9,188	25.5
Atherosclerosis (440) (I70)	1,979	14.8	4,029	25.5	1,055	15.3	7,063	19.6
Surgical operations and other surgical procedures (E878-E879) (Y83-Y84)	967	7.2	847	5.4	1,951	28.3	3,765	10.4
Complications of surgical and medical care ^b^	783	5.9	695	4.4	1,804	26.2	3,282	9.1
Ischaemic heart diseases (410–414) (I20-I25)	930	7.0	1,008	6.4	785	11.4	2,723	7.5
Other heart diseases ^c^	936	7.0	848	5.4	646	9.4	2,430	6.7
Renal failure (584–586) (N17-N19)	559	4.2	648	4.1	809	11.7	2,016	5.6
Cardiorespiratory arrest (427.5. 799.1) (I46.9. R09.2)	417	3.1	679	4.3	604	8.8	1,700	4.7
Heart failure (428) (I50)	399	3.0	387	2.4	427	6.2	1,213	3.4
Chronic lower respiratory diseases (490–496) (J40-J47)	283	2.1	517	3.3	355	5.1	1,155	3.2
Multiple organs failure (799.8) (R68.8)	286	2.1	450	2.8	348	5.0	1,084	3.0
Diabetes mellitus (250) (E10-E14)	361	2.7	414	2.6	264	3.8	1,039	2.9
Cerebrovascular diseases (430–438) (I60-I69)	364	2.7	260	1.6	276	4.0	900	2.5
Other diseases of arteries. arterioles and capillaries (442–448) (I72-I78)	143	1.1	332	2.1	372	5.4	847	2.3
Other respiratory diseases of the interstitium (514–516. 518.5) (J80-J84)	317	2.4	234	1.5	182	2.6	733	2.0
Pneumonias (480–486) (J12-J18)	161	1.2	176	1.1	274	4.0	611	1.7
Respiratory failure. not elsewhere classified (786.0) (J96)	152	1.1	171	1.1	267	3.9	590	1.6
Septicemias (038) (A40-A41)	104	0.8	163	1.0	237	3.4	504	1.4
Neoplasms (140–239) (C00-D48)	110	0.8	241	1.5	142	2.1	493	1.4
Tobacco use [smoking] (305.1) (F17)	124	0.9	200	1.3	105	1.5	429	1.2
Vascular disorders of intestine (557) (K55)	84	0.6	114	0.7	205	3.0	403	1.1
Senility (797.0) (R54)	77	0.6	185	1.2	120	1.7	382	1.1
Other respiratory diseases ^d^	152	1.1	106	0.7	115	1.7	373	1.0
Marfan's syndrome (759.8) (Q87.4)	16	0.1	6	0.0	3	0.0	25	0.1

Source: Fundação Sistema Estadual de Análise de Dados – SEADE.

Rubrics and codes of the International Classification of Diseases. Injuries and Causes of Death. Ninth Revision (1985 to 1995).

Rubrics and codes of the International Statistical Classification of Diseases. and Related Health Problems, Tenth Revision (1996 to 2009).

Data for 1996 are partial.

^a^ Haemorrhages (285.1-.9, 423.0 .9, 459.0, 511.8, 568.8, 578) (D62, D64, I31.2. .9, J94.2, K66.1, K92.0- .2, R58).

^b^ Complications of surgical and medical care (429.4, N996-N999) (E89.9, G97, I97, J95, K91, N99, T80-T88).

^c^ Other heart diseases (390–398, 415–417, 420–422, 423.1-.8, 424.0-.4, 427.6-.9, 429.0-.3, .5-.9 ) (I00-I09, I26-I28, I30-I31, I31.3-.8, I33-I42, I44.0-I46.1, I47-I49, I51).

^d^ Other respiratory diseases (460–478, 487, 500–511.1, 511.9-513.1, 518.0-.4, 518.8-.9) (J00-J11, J20-J39, J60-J70, J85-J94.1, J94.8-9, J98).

### Underlying causes of death with aortic aneurysm and dissection as an associated cause

Underlying causes in the deaths in which aortic aneurysm and dissection were listed as an associated cause are presented in Table 5, according to the ICD structure and also ordered after their total mentions. Major underlying causes of death were included in ICD chapters of diseases of the circulatory, respiratory, digestive systems and neoplasms. Ischemic heart diseases were most frequently reported as underlying causes with dissection and aortic aneurysms without rupture, hypertensive diseases with dissection and ruptured aneurysms and neoplasm with aortic aneurysms without rupture.

**Table 5:** Underlying causes-of-death in deaths that included aortic aneurysm and dissection as associated cause-of-death, State of São Paulo, 1985-2009

**Underlying causes of death** (**ICD**-**9**) (**ICD**-**10**)	**Aortic aneurysm and dissection as associated cause**-**of**-**death**							
	**Dissection**		**Ruptured**		**Without rupture**		**Total**	
	**n**	**%**	**n**	**%**	**n**	**%**	**n**	**%**
**Certain infeccious and parasitic diseases** (**001**–**138**) (**A00**-**B99**)	**23**	**3**.**2**	**14**	**1**.**2**	**122**	**2**.**6**	**159**	**2**.**4**
Septicemias (038) (A40-A41)	12	1.6	7	0.6	54	1.2	73	1.1
Chagas' disease (086) (B57)	3	0.4	5	0.4	19	0.4	27	0.4
**Neoplams** (**140**–**239**) (**C00**-**D48**)	**21**	**2**.**9**	**46**	**3**.**9**	**442**	**9**.**5**	**509**	**7**.**8**
Malignant neoplams of oesophagus (150) (C15)	1	0.1	11	0.9	91	2.0	103	1.6
Malignant neoplasm of bronchus and lung (162.2-162.9) (C34)	5	0.7	7	0.6	72	1.6	84	1.3
**Endocrine**. **nutritional and metabolic diseases** (**240**–**279**) (**E00**-**E90**)	**19**	**2**.**6**	**76**	**6**.**5**	**131**	**2**.**8**	**226**	**3**.**5**
Diabetes mellitus (250) (E10-E14)	8	1.1	41	3.5	72	1.6	121	1.9
**Diseases of the circulatory system** (**390**–**459**) (**I00**-**I99**)	**469**	**64**.**4**	**621**	**53**.**3**	**2**,**548**	**55**.**0**	**3**,**638**	**55**.**7**
Hypertensive diseases (401–404) (I10-I13)	134	18.4	216	18.5	336	7.3	688	10.5
Ischaemic heart diseases (410–414) (I20-I25)	146	20.1	166	14.2	1,067	23.0	1,379	21.1
Pulmonary heart diseases and diseases of pulmonary circulation (415–417) (I26-I28)	14	1.9	9	0.8	65	1.4	88	1.3
Cardiomyopathy (425) (I42)	18	2.5	26	2.2	147	3.2	189	2.9
Other cardiac arrhythmias (426–427) (I44-I49)	6	0.8	11	0.9	58	1.3	75	1.1
Heart failure (428) (I50)	13	1.8	20	1.7	148	3.2	181	2.8
Other heart diseases (420–424. 429) (I30-I40. I51)	37	5.1	25	2.1	161	3.5	223	3.4
Cerebrovascular diseases (430–438) (I60-I69)	67	9.2	108	9.3	387	8.4	562	8.6
Atherosclerosis (440) (I70)	11	1.5	19	1.6	71	1.5	101	1.5
Other diseases of arteries. arterioles and capillaries (442–448) (I72-I78)	15	2.1	12	1.0	68	1.5	95	1.5
**Diseases of the respiratory system** (**460**–**519**) (**J00**-**J99**)	**74**	**10**.**2**	**129**	**11**.**1**	**696**	**15**.**0**	**899**	**13**.**8**
Pneumonias (480–486) (J12-J18)	31	4.3	44	3.8	270	5.8	345	5.3
Chronic lower respiratory diseases (490–496) (J40-J47)	21	2.9	52	4.5	302	6.5	375	5.7
**Diseases of the digestive system** (**520**–**579**) (**K00**-**K93**)	**49**	**6**.**7**	**182**	**15**.**6**	**348**	**7**.**5**	**579**	**8**.**9**
Vascular disorders of intestine (557) (K55)	3	0.4	19	1.6	86	1.9	108	1.7
Peritoneal adhesions (568.0) (K660)	24	3.3	121	10.4	10	0.2	155	2.4
**Diseases of the geniturinary system** (**580**–**629**) (**N00**-**N99**)	**12**	**1**.**6**	**17**	**1**.**5**	**174**	**3**.**8**	**203**	**3**.**1**
Renal failure (584–586) (N17-N19)	5	0.7	10	0.9	118	2.5	133	2.0
**Pregnancy**. **childbirth and the puerperium** (**630**–**676**) (**O00**-**O99**)	**4**	**0**.**5**	**3**	**0**.**3**	**1**	**0**.**0**	**8**	**0**.**1**
**Congenital malformations**. **deformations and chromos**… (**740**–**759**) (**Q00**-**Q99**)	**27**	**3**.**7**	**16**	**1**.**4**	**23**	**0**.**5**	**66**	**1**.**0**
Marfan's syndrome (759.8) (Q87.4)	24	3.3	10	0.9	18	0.4	52	0.8
**External causes of morbidity and mortality** (**800**–**999**) (**V00**-**Y98**)	**11**	**1**.**5**	**43**	**3**.**7**	**41**	**0**.**9**	**95**	**1**.**5**
**Other underlying causes of death**	**19**	**2**.**6**	**18**	**1**.**5**	**108**	**2**.**3**	**145**	**2**.**2**
**Total**	**728**	**100**.**0**	**1**,**165**	**100**.**0**	**4**,**634**	**100**.**0**	**6**,**527**	**100**.**0**

Source: Fundação Sistema Estadual de Análise de Dados – SEADE.

Rubrics and codes of the International Classification of Diseases. Injuries and Causes of Death, Ninth Revision (1985 to 1995).

Rubrics and codes of the International Statistical Classification of Diseases and Related Health Problems, Tenth Revision (1996 to 2009).

### Seasonal variation

**Figure 5:**
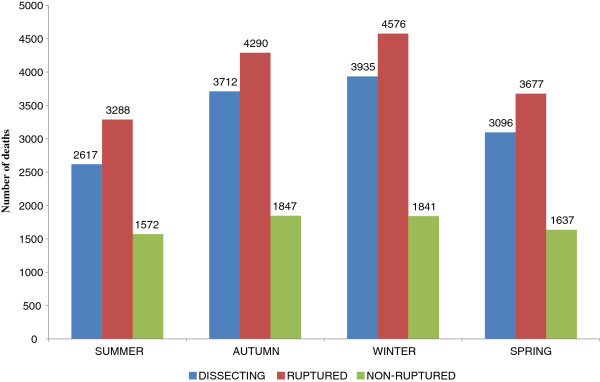
**Number of deaths related to aortic aneurysm**, **ruptured non**-**ruptured and aortic dissection as the underlying cause of death**, **according seasons of the year**, **state of São Paulo**, **1985**–**2009.**

The seasonal variation of aortic dissection, ruptured and non-ruptured aortic aneurysms identified as underlying-causes-of-death are shown in Figure 5. A significant seasonal variation, with highest frequency during winter, occurred in the deaths of aortic dissection (*x*^*2*^ 321.759, *df* 3, *p*<0.0001), ruptured aneurysms (*x*^*2*^ 257.724, *df* 3, *p*<0.0001) and aneurysms without mention of rupture (*x*^*2*^ 34.502, *df* 3, *p*<0.0001). All comparisons between each season with other ones, two by two, for dissection, ruptured and non-ruptured aneurysms resulted significant, except for the deaths of non-ruptured aneurysm that occurred during summer and spring (*x*^*2*^ 1.317, *df* 1, *p=*0.2512) and autumn and winter (*x*^*2*^ 0.01, *df* 1, *p=*0.9213). Similarly, these data are presented by means of proportional mortality in Table 6, which once more establishes highest percentages of deaths mainly in winter for aortic ruptured aneurysms and aortic dissection.

**Table 6:** Proportional mortality (%) related to aortic aneurysms, ruptured and non-ruptured, and aortic dissection, as underlying cause-of-death, according to seasons of the year, state of São Paulo, Brazil, 1985 to 2009

**Variables**	**Summer**	**Autumn**	**Winter**	**Spring**	**p value**
Dissection	0.1987	0.2587	0.2610	0.2302	<0.00001
Ruptured	0.2497	0.2990	0.3035	0.2734	<0.00001
Non-ruptured	0.1194	0.1287	0.1221	0.1217	0.16265

### Crude death rates according to Health Directions of the state

**Figure 6:**
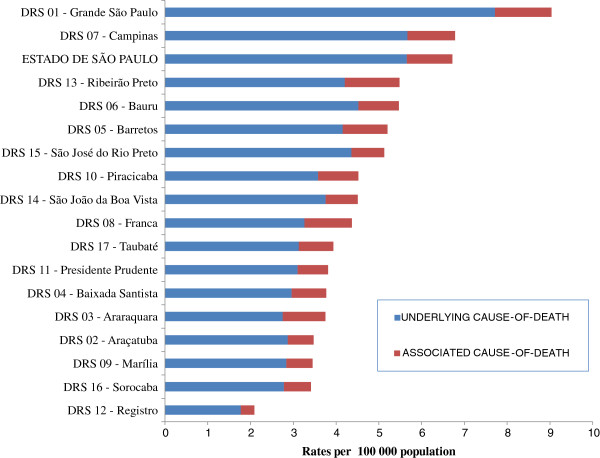
**Age**-**standardized death rates related to aortic aneurysm and dissection according to Regional Health Directions**, **state of São Paulo**, **averaged from 1985 to 2009.**

Figure 6 shows the age-standardized death rates related to aortic aneurysm and dissection by the 17 Regional Health Directions of the state of São Paulo averaged for the entire period from 1985 through 2009. The bars of the figure are formed by the sum of underlying and associated causes rates whose total values correspond to the rates for total mentions of aortic aneurysm and dissection on death certificates. The areas of Grande São Paulo and Campinas appear with rates higher than the average state rate, followed by Ribeirão Preto, Bauru, Barretos and São José do Rio Preto, which are developed areas that also hold some of the most important universities and medical centers of the state. Notable rate variations can be seen, from the highest one of 9.0 per 100,000 population in Grande Sao Paulo to the lowest figure of 2.1 per 100,000 population in Registro, considering total mentions of causes listed on death certificates.

### Number of causes mentioned per death certificate

There was a trend of increasing mean numbers of causes mentioned on the death certificate from 1985–1995 to 1996–2009 subperiods. These numbers were always lower for dissection and aortic aneurysm as the underlying cause, respectively 2.93 (± 1.10) and 3.33 (± 1.33) than on the corresponding death certificates as an associated cause 4.05 (± 1.07) and 4.48 (± 1.17); and also lower for death certificates in women, respectively 3.04 (±1.14) and 3.43 (±1.35) than on those ones of men 3.10 (±1.17) and 3.56 (±1.38) for total mentions of causes (Table 2).

## Discussion

One of the most favorable findings of our study was the observation that around 85% of deaths related to aortic aneurysm and dissection are identified as the underlying cause-of-death, proportion that specifically for ruptured aneurysms reaches 93%, and for non-ruptured aneurysms 60%, enlightening the burden of these deaths on primary mortality statistics. These percentages are superior to those ones found in the United States during 1979–1982 [11], 1980, 1990 and 1998 [44] and 2000–2001 [45], varying from 70% to 72% for the total mentioned deaths, around 90% for dissection, and 93% for ruptured aneurysms, however of 37% for non-ruptured aneurysms [11]. In a similar way, the alternative comparison of the ratios found of mentioned to underlying cause related to ruptured abdominal aortic aneurysm (1.08) and non-ruptured ones (1.75) and the corresponding ratios of England, Oxford region, respectively 1.02 and 1.95 [13], emphasize the importance of these data. Aortic aneurysm and dissection almost invariably appear on death certificates as underlying cause-of-death owing to their high fatality rates and near immediate death after rupture [46].

It may be argued how much actual aortic aneurysm and dissection are assumed as a cause-of-death. i.e., in other words, how trustworthy are mortality statistics. Few studies took care of the question. An unpublished review of the Mayo data disclosed that over 85% of Rochester residents diagnosed with aortic aneurysms that died between 1951 and 1980 had on their death certificate an aortic aneurysm as the underlying cause-of-death, and almost all based on radiographic findings [8]. In England, Oxford region, during 1979 and 1987, a linkage study between hospital and death certificate records disclosed that 86.5% of the people who died within four weeks of hospital admission with an aortic aneurysm as the main diagnosis included it mentioned on death certificates and among these ones 92.5% were identified as an underlying cause-of-death [47].

We observed trends of increased age-adjusted mortality rates related to aortic aneurysm and dissection in the state of São Paulo (both ruptured and non-ruptured), while leveling off and non-significant decreases occurred, mainly in men and as associated (non-underlying) causes of death, from 1996/2004 until 2009. The aging of the Brazilian population is one of the main factors that explain these rising rates; life expectancy in São Paulo between 1980 and 2009 rose from 63.3 to 71.3 years for men and from 70.0 to 78.5 years for women. Also, growing awareness of the disorder, better access to medical services and continuous improvement of mortality statistics contribute to these results. However, a definite increase of the incidence of aortic aneurysm and dissection should not be disregarded. The successive yearly increases of death rates observed in older groups in our study securely support this hypothesis. Advancing age is an important risk factor related to aneurysm enlargement. Older patients who previously died in consequence of other conditions, mostly cardiac disease, are now susceptible of being a victim of aneurysm growth and rupture [41].

While an increased trend was found in the state of São Paulo, decreases of age-standardized mortality rates of aortic aneurysm and dissection as underlying and all mentions of causes were verified in England from 1996 and 2004 [15] and in the United States, for aortic aneurysm as underlying cause-of-death, from 1990 to 2006 [48]. Concomitantly, recent evidence have shown significant decreasing age-adjusted mortality trends from abdominal aortic aneurysm in Australia (1999 to 2006), New Zealand (1991 to 2007), England &Wales and Scotland (1997 to 2009), England & Wales (2001 to 2009), and in the United States (1979 to 2007) [49-54]. The factors associated with mortality changes related to aortic aneurysm and dissection are complex [52,53]. In the state of São Paulo, the increasing longevity of Brazilian population is likely to have contributed with the mortality rise since 1985.

Older ages at death in women reflects their greater life expectance and higher male mortality related to aortic aneurysm and dissection. Similarly, older ages in the deaths related with aortic aneurysms and dissection as associated (non-underlying) causes results from the predominance of non-ruptured aneurysms (71.0%), with better survival rates and minor fatality rates. Mean age in patients with intact AAA increased over time, whereas no significant age trend was observed in those with ruptured AAA [55]. The finding of lower mean ages at death for dissections (63.2%), in comparison to aneurysm disease with or without mention of rupture (68.4 and 71.6% respectively), can be explained, in spite of the limitations regarding the reference of dissection as type A or B rarely being certified as cause-of-death and its absence in the structure of the International Classification of Diseases. Given that type A accounts for 62% of cases of dissections, that are significantly more common in younger individuals, and with higher fatality rates [40], the observed lower mean age at death can be understood.

Age-standardized death rates related to aortic aneurysm and dissection were always higher in men than in women, however the difference between these rates has become smaller during the study period. This tendency has also been verified in other countries [56]. Among the factors connected with this trend in women are the higher incidence of aortic aneurysm and dissection resulting from older age, high prevalence of hypertension and current smokers, and the greater mortality and fatality risks [57-59]. The mean age at death of women was 1.45 years higher than of men in 1985 and increased to 3.41 years in 2009.

A significant mortality difference between men and women in relation to causes-of death was observed. Deaths related to abdominal aortic aneurysm and dissection prevail among men whereas aneurysms of unspecified site and dissections are more frequent in women. Along with the facts that aneurysms of unspecified site are an inaccurate cause-of-death and that their majority is considered to be abdominal aortic aneurysms, to our knowledge, there are no plausible explanations for their prevalence in women. In England and Wales, from 1974 to 1984, the type was not specified for one quarter of aneurysm deaths [9]. More research is desired to clarify the questions related to this difference.

The aggregate of hemorrhages is the main associated cause of the deaths related to overall aortic aneurysm and dissection, indicating the occurrence of a rupture. A rupture may have even happened in those 14% of aneurysm without such mention owing to omitted data on the death certificates. Hemorrhage was also one of the most common associated causes of aortic aneurysm among deaths in a cohort of men employed at eight oil refineries in the Unite Kingdom [15]. Hemorrhages are considered important predictors of death [60]. Another critical issue to be taken into account aiming at the property of mortality statistics is the link of hemorrhages and ruptured aneurysms in decision tables during automated processing of causes of death.

Surgical operations and procedures and its complications were mentioned in death certificates among the associated causes of death, mainly for non-ruptured aneurysms. Venous and arterial iatrogenic injuries have been shown to occur as a negative impact on the survival, worsening the effects of hemorrhages and shock [60].

Our finding that hypertension is the main associated (non-underlying) cause in deaths from dissections (present in 49% of the studied group) is consistent with other studies which have shown that 70% of patients diagnosed with aortic dissection present elevated blood pressure on admission. The prevalence of hypertension in Brazil is very high, accounting, in 1996, around 21.6% (24.4% in women and 18.4% in men) and for those aged 65 years and over 57.7% (61.5% in women and 51.7% in men) [61]. This fact may explain the high mortality related to dissection found in São Paulo. High blood pressure levels may influence atheroma formation and increase hemodynamic stress involved in aneurysm rupture and dissection, emphasizing the importance of simple prevention measures focusing on mortality reduction.

We have shown strong association among aortic aneurysm and dissection as underlying and hypertension and atherosclerosis as non-underlying causes. This association was also evaluated by means of ratios of the observed to expected age standardized joint frequencies of these causes irrespective of their qualification, using the leading causes of death in the United States. For 1980, the pairwise ratio between aortic aneurysm/dissection and hypertension was 1.87 and between aneurysm/dissection and atherosclerosis 1.11. Also, the joint frequency of three-way combination of these causes of death disclosed a ratio of observed to expected of 3.40. Ratios greater than 1 specify an association and greater than 2 are considered large [44].

Our study is the first one to consider underlying causes for aortic aneurysm and dissection as an associated cause-of-death, 71.0% non-ruptured ones, using population based mortality data. While not fully comparable, equivalent results of a study with non-ruptured abdominal aortic aneurysms which underwent surgical repair has shown ischemic heart diseases, neoplasms and respiratory system diseases as the main underlying causes of death after hospital discharge and >30 days [62]. At older ages, these are the leading cause-of-death of the corresponding populations in Brazil and United States.

The association observed between aneurysms without rupture and neoplasm as an underlying cause-of-death emphasize how screening is an important tool for early detection and mortality reduction. Screening programs can reduce up to 48-55% mortality rates related to aneurysm and still be cost-effective when applied to a pre-determined risk population. Since oncologic disorders are not considered risk factors for aneurysm development, the growing referral of oncologic patients to vascular services reflects how the general population is clearly under-diagnosed.

Evidence suggesting a seasonal variation in the incidence of rupture of aortic aneurysms and dissection has been reported, with peak values occurring during autumn and especially during winter months [63-65]. In our study we were able to present a seasonal variation, with peaks in winter months, of aortic dissection, ruptured and non-ruptured aortic aneurysms identified as underlying-causes-of-death. In England and Wales, from 1991 to 1995, an evident peak in winter also occurred for recorded deaths from ruptured abdominal aortic aneurysms [56]. Seasonal changes in São Paulo, located in the southeastern region of Brazil, are better defined and the incidence of rupture correlates with the expected pattern, with significantly higher death incidence noted in the colder months of May to September. Increased blood pressure levels, arterial spasm, pulmonary disease exacerbations and passive smoking in colder weather can explain this pattern, also observed in other cardiovascular conditions such as cerebral and coronary ischemia [66].

Population mortality statistics suffer from quantity and quality problems. In the state of Sao Paulo there is full coverage of deaths [67]. Regarding quality, ill-defined causes amount about 6% of total death, corresponding to less than those reported for the entire country [68-70]. Besides, coding and automated processing of causes of death continue under rigorous quality control [18]. All death certificates are filled by physicians and the verified mean number of causes mentioned by death certificate rank among the highest in the world. While ICD-9 was in force and Part I of the International Form of Medical Certificate of Cause-of-death still included only three lines, from 1985 to 1995, in São Paulo, the overall mean number of causes per aortic aneurysm and dissection death certificates was 3.08. This average is superior to the corresponding mean numbers of 2.19, 2.25 and 2.23 observed in the United States in 1980, 1990 and 1998 in aged 65 and over [44], and the mean number of 2.75 found in selected jurisdictions of Canada from 1990 to 1993 [46].

However, specific limitations occur with aortic aneurysm and dissection as a mentioned cause-of-death. An abdominal, thoracic or of unspecified site aortic aneurysm may be informed as an "dissecting aneurysm" by physicians when completing the death certificate; the "dissecting" adjective may lead the cause-of-death to be understood as being a dissection instead of an aneurysm. Therefore, the observed values of dissection mortality rates may be overestimated. Another concern is the high proportion of aneurysm of unspecified site appearing as a cause-of-death, second and third most frequent sites respectively among women and men in our study. Classification of aneurysm by anatomic site is an important data considering that different sites may be associated with variations in their natural history, clinical presentation and means of treatment [71]. When completing death certificates, physicians should pay attention to inform correctly the aneurysm site. Otherwise, statistical offices that cope with population mortality data should query death certificates including an aneurysm unspecified site, similar to procedures with ill-defined causes-of-death. Finally, mortality statistics may underestimate aortic aneurysm and dissection. Patients submitted to successful surgical treatment or patients with unsuspected disease are not accounted for in this type of evaluation. Moreover, when the patient dies from ruptured aneurysm or dissection in the first moments after hospital admission or medical assistance at home, the symptoms of chest/abdominal pain, syncope and paleness may be interpreted by the physician as myocardial infarction, before any image tests can be performed. Abdominal aortic aneurysms may be difficult to detect by palpation in obese patients.

## Conclusion

Given the significance of aortic aneurysm and dissection as a leading cause-of-death, it is important to accurately document epidemiologic trends in order to plan effective measures aiming mortality reduction. In Brazil, epidemiologic studies on aneurysms are scarce and the available literature is outdated. Thus the relevance of this research to (1) analyze the progression of the disease in Brazilian population (2) propagate knowledge within medical community (3) enable statistical comparisons with the western world (4) estimate future trends and orientate government investments.

## Competing interests

The authors declare that they have no competing interests.

## Authors’ contributions

AHS participated in the design of the study, processing, analysis and interpretation of data and drafted the manuscript. PP-L and MK have been involved in drafting and critically revising the manuscript for important intellectual content. All authors read and approved the final manuscript.

## Pre-publication history

The pre-publication history for this paper can be accessed here:

http://www.biomedcentral.com/1471-2458/12/859/prepub
